# Effect on Multipotency and Phenotypic Transition of Unrestricted Somatic Stem Cells from Human Umbilical Cord Blood after Treatment with Epigenetic Agents

**DOI:** 10.1155/2016/7643218

**Published:** 2015-12-14

**Authors:** Foued Ghanjati, Simeon Santourlidis

**Affiliations:** Institute for Transplantation Diagnostics and Cell Therapeutics, Heinrich Heine University, 40225 Düsseldorf, Germany

## Abstract

The epigenetic mechanism of DNA methylation is of central importance for cellular differentiation processes. Unrestricted somatic stem cells (USSCs) from human umbilical cord blood, which have a broad differentiation spectrum, reside in an uncommitted epigenetic state with partial methylation of the regulatory region of the gene coding for the pluripotency master regulator OCT4. Thus we hypothesized that further opening of this “poised” epigenetic state could broaden the differentiation potential of USSCs. Here we document that USSCs drastically change their phenotype after treatment by a new elaborated cultivation protocol which utilizes the DNA hypomethylating compound 5′-aza-2-deoxycytidine (5-Aza-CdR) and the histone deacetylase inhibitor trichostatin A (TSA). This treatment leads to a new stable, spheroid-forming cell type which we have named SpheUSSC. These cells can be stably propagated over at least 150 cell divisions, express OCT4, retain the potential to undergo osteogenic differentiation, and have additionally acquired the ability to uniformly differentiate into adipocytes, unlike the source USSC population. Here we describe our treatment protocol and provide evidence that it induces a dedifferentiation step and concomitantly the acquisition of an extended differentiation capability of the new SpheUSSC type.

## 1. Introduction

Adult multipotent stem cells from human umbilical cord blood are a promising cell source for a variety of stem cell replacement therapies in regenerative medicine. They are able to self-renew, have a high proliferative rate, and possess the potential to differentiate into specialized cells. Multipotent stem cells, as, for example, adult human mesenchymal stem cells (MSCs) from bone marrow, can differentiate into various lineages of mesenchymal tissues, including bone, cartilage, fat, tendon, muscle, and marrow stroma [1]. Unrestricted somatic stem cells (USSCs), another multipotent stem cell population from human cord blood, share the osteogenic and chondrogenic differentiation pathway with cord blood MSCs but are unable to undergo adipogenic differentiation, in accordance with their strong expression of the adipocyte inhibitor DELTA, HOMOLOG-LIKE 1/PREADIPOCYTE FACTOR 1 (DLK-1/PREF1) [2].

Despite the broad-ranging evidence for a key role of epigenetics in embryonic stem cell differentiation, epigenetic mechanisms and in particular the role of DNA methylation in adult multipotent stem cells are less well investigated [3]. Interestingly, it has been reported that a certain subpopulation of human umbilical cord blood cells is able to acquire OCT4 and NANOG expression and the ability to differentiate into all three germ layers after undergoing an epigenetic partial reprogramming which had been induced by cultivation in FSFI medium [4]. However, it is only partly understood which specific epigenetic modifications control the maintenance of multipotency and determine the differentiation options of multipotent stem cells. In fact, on the basis of recent evidence [5, 6], it is intensively discussed whether CpG methylation of specific lineage gene promoters may restrict lineage differentiation of multipotent stem cells and, furthermore, whether the removal of these epigenetic marks could relieve these restrictions to provide a broader differentiation spectrum of adult multipotent cells [3]. Indeed, treatment of multipotent bone marrow MSCs with the DNA demethylating agents 5-aza-2′-deoxycytidine (5-Aza-CdR) or 5′-azacytidine (Aza) induces a transition towards the osteogenic lineage [7, 8] and BM-MSCs treated with both 5-Aza-CdR and the histone deacetylating reagent Trichostatin A (TSA) undergo neural differentiation [9]. Notably, we have observed that the concentration of such epigenetic inhibitors has to be adjusted to each cell type in order to avoid enhancement of cell death but to still induce an epigenetic response [10, 11]. Moreover, despite a marked induction of transcription, the affected gene promoters become in most cases only partially demethylated, retaining an intermediate methylation pattern, as observed, for example, by Arai et al. [12]. In USSCs we had accordingly observed a partially methylated 5′ region of OCT4 [13], reminiscent of the state in trapped reprogrammed iPSC described by Mikkelsen et al. [14]. Consequently, we hypothesized that a further relaxation of the epigenetic state might entail effects on USSC multipotency by, among others, activation of the pluripotency key regulator OCT4. In this study we describe an elaborated treatment of USSCs with epigenetic drugs leading to a marked and stable change of the cellular phenotype and differentiation behavior.

It is generally accepted that epigenetic gene regulation mechanisms are of fundamental importance for cellular differentiation and reprogramming towards pluripotency [15]. This has been convincingly demonstrated by various reprogramming techniques applied on somatic cells, including somatic nuclear transfer into enucleated oocytes, ES cell fusion with somatic cells, and induction of pluripotency by defined pluripotency factors. For instance, the generation of induced pluripotent stem cells (iPSC) goes along with a highly dynamic epigenetic transition from the differentiated state to pluripotency, during which previously developmentally established epigenetic marks are overridden or erased [14]. Fully reprogrammed cells acquire a genome-wide “open” chromatin state, characterized by H3K4me3 enrichment and DNA hypomethylation, similar to that of embryonic stem cells. Concomitantly, reactivation of key pluripotency factors like OCT4, NANOG, and REX1 takes place which is preceded by demethylation of their gene promoters [14].

In contrast, partially reprogrammed cell states appear trapped in an intermediate stage of the reprogramming process that is characterized by incomplete epigenetic reversion, including residual methylation of key pluripotency factors. However, it has been demonstrated that this situation can be resolved in part by DNA methyltransferase inhibitors, for example, 5-azacytidine (Aza), improving the overall efficiency of the reprogramming process [14].

## 2. Material and Methods

### 2.1. Protocol of Generation of SpheUSSCs by Treatment of USSC SA 8/25 with 5-Aza-CdR and TSA

**Figure 1:**
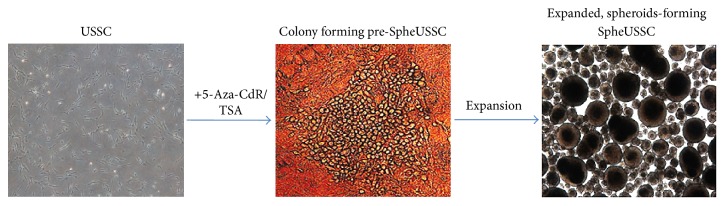
USSCs treated with the epigenetic agents 5-Aza-CdR and TSA stepwise change their morphology into a new spheroids-forming cell type named SpheUSSCs. The treatment of USSCs with 5-Aza-CdR and TSA first alters the USSC phenotype into a flat colony-forming cell type (pre-SpheUSSC) which after separation by trypsine and splitting and expansion of the cells alters into the stable, spheroids-forming cell type SpheUSSC.

Umbilical cord blood was collected as previously described with the informed consent of the mothers [16]. Umbilical cord blood derived USSCs (SA 8/25 passage 6 (p6), SA 5/73 (p6)) and MSC cells were cultivated as described and kindly provided by Dr. Kögler et al. [16]. In total four cord blood stem cell lines were tested. Trypsinized USSCs and MSCs of 6 passages, each with 100 × 10^3^ cells, were separately plated in one well of a 6-well plate and cultivated until 60 to 70% of confluence. At this point the USSC medium was completely replaced by induction medium, which consists of DMEM, 20% FCS, 2 mM L-glutamate, 0,1 mM *β*-mercaptoethanol, 1% penicillin/streptomycin, 100 ng/mL recombinant Human Stem Cell Factor, and freshly prepared 1 *μ*M 5-Aza-CdR and 25 nM TSA. These working concentrations were determined after serial dilutions. They lead to a minimum of cytotoxicity by still providing an epigenetic effect on the cells. Other epigenetic drugs were not tested. Then the epigenetic drugs were replaced by freshly prepared ones in the mentioned concentrations every 24 hours for three days. At day 4, 50 to 100 × 10^3^ of these epigenetically modified cells were transferred into a new well of a 6-well plate and propagated in fresh USSC medium without epigenetic drugs. Medium exchange occurred every 3 days. After 10 to 14 days, the first prespheroid colonies appeared. A representative one is illustrated in the middle of Figure 1. Individual colonies were picked up by a sterile pipette tip and transferred into a 6 cm plate containing 3 mL of USSC medium with thorough resuspension. Then after every 48 h of incubation the medium was removed and replaced by a new expansion medium consisting of 85% of basal medium (390 mL KnockOut DMEM/F-12 (Gibco), 100 mL KnockOut serum replacement (Gibco), 5 mL nonessential amino acids, 5 mL Pen/Strep/Glu, and 100 *μ*M *β*-Mercaptoethanol) and 15% of conditioned medium, collected shortly before this passage. A new cell type arises after 10 days which organizes itself in spheroids of varied sizes of 40 to 270 *μ*m within the next 5–7 days of incubation (Figure 1). Spheres were dissociated with trypsin, resuspended in DMSO, and transferred into liquid nitrogen for freezing.

### 2.2. Differentiation of SpheUSSCs into Adipocytes and the Osteogenic Lineage

For adipocyte differentiation, SpheUSSCs were cultivated in expansion medium as described above with medium renewal every 3 days without passaging. After 7 days the cells become sessile and begin to differentiate. After 10 days, the first small lipid droplets are visible within the cells which gradually increase in number until day 14. After 3 weeks the differentiation process is completed, since >95% of all cells are no longer dividing, harbor many lipid droplets of varied sizes, and resemble adipocytes. This adipogenic differentiation has been performed three times with SpheUSSCs.

For osteogenic differentiation, SpheUSSCs were cultivated in the following osteogenic optimized and ready to use Osteogenic Differentiation Medium, SH30881 (Thermo Scientific, USA). After 7 days, thin and white sediments resemble calcium deposits appeared on the cells. After 14 days all the cells ceased proliferation and were covered by white sediments. This osteogenic differentiation has been performed two times with SpheUSSCs.

### 2.3. Alizarin Red S Staining and Oil Red O Staining

**Figure 2:**
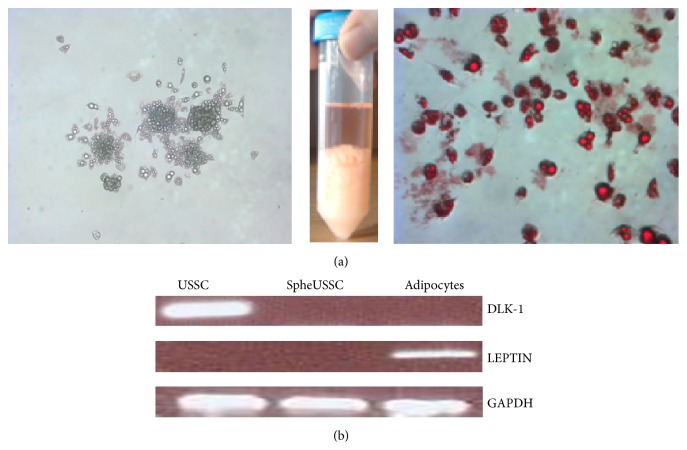
SpheUSSCs can be spontaneously and uniformly differentiated into multivacuolar adipocytes. After cultivation with expansion medium unto the spatially confined cell expansion maximum is reached, SpheUSSCs first ceased proliferation and become sessile and then they uniformly develop multivacuolar adipocytes ((a), left and right panel), which can be stained by Oil Red O Solution ((a), right). From one middle sized cultivation flask of 75 cm^2^ the amount of fat shown in the middle of figure's part (a) can be isolated. Repression of transcription of the adipogenesis inhibiting factor* DLK-1* accompanies the generation of SpheUSSCs and persists during adipogenic differentiation, which in addition is characterized by acquisition of* LEPTIN* transcription (b).

**Figure 3:**
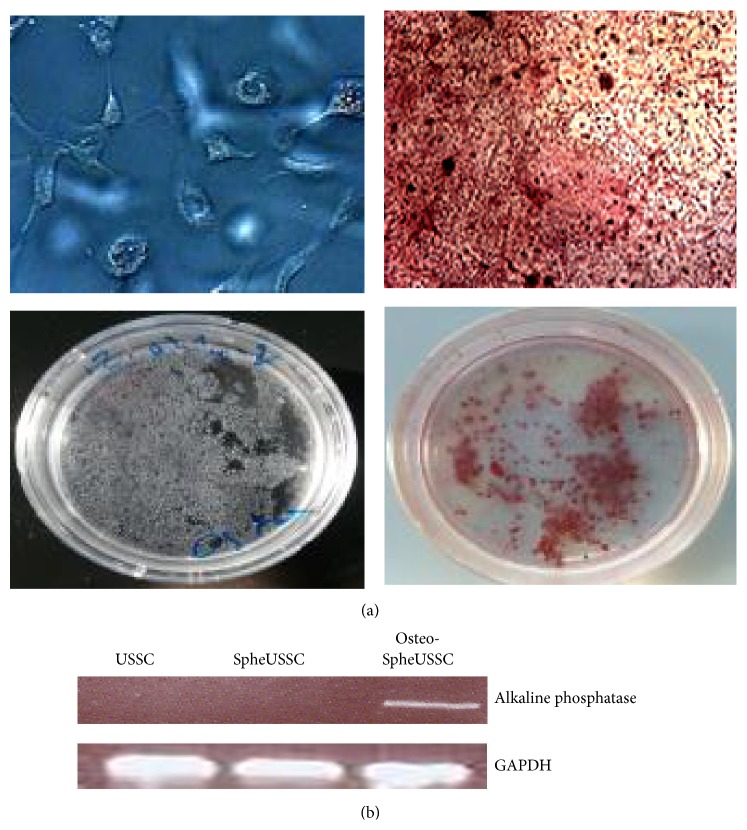
SpheUSSCs possess osteogenic differentiation potential. SpheUSSCs can undergo a rapid and uniform osteogenic differentiation ((a), both panels on the left) with calcium deposits as were detected with Alizarin Red S staining ((a), both panels on the right). Reverse transcription PCR detects transcription of* alcaline phosphatase* gene (b).

The adipocytes were derived as described above. After 3 weeks, lipid droplets were assessed by oil red O staining, as suggested in the manufacturer's protocol (Sigma-Aldrich, St. Louis, MO, USA). The stained lipid droplets were investigated with an inverted phase-contrast microscope (Axio Vision, Release 4.7, Carl Zeiss Imaging Solutions GmbH, Hallbergmoos, Germany) (Figure 2). Osteogenic differentiation was assessed by alizarin red S staining for the presence of calcium deposits as suggested by the manufacturer (Sigma-Aldrich, St. Louis, MO, USA). The cells were first fixed with 4% formaldehyde (Sigma-Aldrich, St. Louis, MO, USA) for 30 min at room temperature, rinsed with distilled water, and then stained with 2% (w/v) alizarin red S (Sigma-Aldrich, St. Louis, MO, USA) dissolved in distilled water (pH 4.2; adjusted with 10% ammonium hydroxide, Sigma-Aldrich, St. Louis, MO, USA) for 45 min. Cells were then washed extensively with distilled water and examined for mineralization of extracellular matrix (ECM) (Figure 3).

### 2.4. RNA Preparation, cDNA Synthesis, and Real-Time PCR

RNA was prepared using the RNeasy Mini Kit (Qiagen) according to the manufacturer's instructions. First-strand cDNA synthesis was performed from 1.5 *μ*g RNA by reverse transcription using oligo(dT) (Promega) and Moloney murine leukemia virus reverse transcriptase (Promega) in a volume of 50 *μ*L at 42°C for 1 h. Real-time PCR was carried out with SYBR Green PCR Mastermix (Applied Biosystems) using 25 ng template cDNA. All reactions were run in triplicates on a StepOnePlus System (Applied Biosystems, Foster City, CA). Standard curves were generated using StepOne software v2.1 (Applied Biosystems). The sequences for the primers were carefully examined and checked for their specificity (Supplementary data, Table  1 in Supplementary Material available online at http://dx.doi.org/10.1155/2016/7643218). Relative changes in gene expression were calculated following the ΔΔCt-method with glyceraldehyde-3-phosphate dehydrogenase (GAPDH) as a standard.

### 2.5. Preparation of DNA

Cellular DNA was isolated from SpheUSSC, USSCs, MSCs, and ESC-H9, using the QIAamp DNA Mini Kit (Qiagen, Hilden, Germany) according to the manufacturer's protocols. This DNA was used for methylated DNA immunoprecipitations (MeDIP) and subsequent NimbleGen (Roche) array application.

### 2.6. Bisulfite Genomic Sequencing

Genomic sequencing of bisulfite-converted DNA was performed as described [10, 11]. In brief, bisulfite conversion was performed using the EpiTect Kit (Qiagen). All PCR primers used in this study are listed in Table  1 of supplementary data.

The amplification conditions were denaturation at 95°C for 13 min, followed by 35 cycles of 95°C for 60 s, TM for 40 s, and 72°C for 30 s. The TA Cloning Kit (Invitrogen) was used for cloning of the amplification products according to the manufacturer's instructions. Sequencing was performed with the BigDye Terminator Cycle Sequencing Kit (Applied Biosystems) on a DNA analyzer 3700 (Applied Biosystems) using M13 primer. On average 30 clones were sequenced to obtain a representative methylation profile from one sample. This experiment was done two times with the SpheUSSC cells with the same result.

### 2.7. Western Blot

USSCs, SpheUSSCs, and ESC-H9 were lysed in lysis buffer containing 5 M NaCl, 1% NP-40, 0.5% DOC, 0.1% SDS, 1 mM EDTA, 50 mM Tris, pH 8.0, and freshly added 10 *μ*L/mL protease inhibitor (Sigma). 15 *μ*g of protein was resolved in a 12% sodium dodecyl sulfate-PAGE gel and transferred onto Immobilon-P membrane (Millipore). Membranes were probed with primary antibody against OCT4 (sc5279, Santa Cruz Biotechnology) at 4°C overnight, washed with 0.1% Tween-20 in Tris-buffered saline, and incubated with secondary antibody conjugated with horseradish peroxidase. The signals were visualized with enhanced luminescence (WesternBright Quantum, Advansta).

### 2.8. MeDIP and Array Analyses of Cellular DNA

One microgram of each DNA fraction was sonicated to yield 300–1000 bp fragment size by the Vibra Cell 75022 Ultrasonic Processor (Novodirect). These DNA samples then underwent immunoprecipitation of methylated DNA employing the Diagenode's MeDIP Kit in accordance with the manufacturer's instructions. Amplification of input and output samples occurred by applying the Genome Plex Complete WGA Kit (Sigma-Aldrich) as described in the user's guide. Hybridization of 1 *μ*g of each amplified DNA sample was performed on NimbleGen 385 K RefSeq Promoter Arrays HG18 containing all known RefSeq genes (Roche). The promoter regions on these arrays are covered by 75 mer probes with approximately 100 bp spacing. The hybridization procedure was applied as suggested by the manufacturer. The hybridized arrays were scanned on an Axon 4000B microarray scanner (Molecular Devices, Sunnyvale CA), and the images were analyzed with Axon GenePix software version 4.1. Image and data analyses were processed with NimbleScan version 2.5 and SignalMap version 1.9.

### 2.9. Telomere Length Assay and Senescence Assay

The measurement of telomere length was performed with TeloTAGGG Telomere Length Assay and according the manufacturer's protocol (Roche Life Science). This experiment was done three times with the same result. For evaluation, a PCR-based independent approach was conducted according to the published method of O'Callaghan and Fenech [17]. Senescence *β*-Galactosidase Staining Kit from Cell Signaling Technology, Inc., was used according the manufacturer's protocol. This experiment was done two times with the same result.

## 3. Results

### 3.1. Generation, Cultivation, and Storage of SpheUSSCs

The USSC line 8/25 [16] exhibits a senescent rate of 30%–45% and can be cultured for up to maximal 63 cumulative population doublings (CPD) [2]. We applied our elaborated epigenetic treatment protocol three times independently on the USSC 8/25 and reproducibly obtained every time uniform, descendant cells which are shown in Figure 1. These cells are stable, divide every 48 h, and can be cultured for at least 1.5 years and at least 150 cell divisions without acquiring any morphological changes or features of senescence. The absence of senescence was repeatedly confirmed throughout the culture period by senescence assay (supplementary data). This new cell type can be repeatedly frozen and thawed, even after having gone through more than 150 cell divisions, without loss of its proliferation and differentiation capabilities. Applying our epigenetic treatment protocol to two MSC lines and another USSC line, named USSC 5/73 (P6), initially led to a generation of an altered cellular phenotype, resembling the flat colony forming pre-SpheUSSCs as shown in Figure 1, but could not be further propagated under the chosen conditions. Thus only one umbilical cord blood derived stem cell line out of four tested was able to develop this new stable, spheroid-forming cell phenotype after treatment by our epigenetic protocol. We named this new cell type SpheUSSC.

### 3.2. SpheUSSCs Are Uniformly Able to Differentiate into Multivacuolar Adipocytes and Undergo Osteogenic Differentiation

While evaluating the properties of SpheUSSCs under various cell culture conditions we made the following observation. Unpassaged SpheUSSCs that had completely filled up the cultivation vial ceased proliferation, became sessile, and spontaneously and uniformly differentiated into multivacuolar cells with evident globules of fat inside the vacuoles, reminiscent of brown adipocytes of newborns, that could be stained by Oil Red O (Figure 2(a)). The staining was uniform over the whole cell population, indicating that every single SpheUSSC had terminally differentiated into a fat cell within 14 days after the SpheUSSCs had become confluent. From one single middle sized culture flask of 75 cm^2^ covered by fat cells we could isolate a huge amount of fat as documented in the center of Figure 2(a). The differentiated cells express leptin (Figure 2(b)), a factor produced by brown and white adipocyte tissue [18], whereas the adipogenesis inhibitor DLK-1, which is expressed in the source USSCs, was undetectable in SpheUSSCs and their adipocyte derivatives (Figure 2(b)). Thus, SpheUSSC can be uniformly differentiated into adipocytes, which was previously not feasible for the 8/25 USSC source cells.

Despite their newly acquired potential for adipocyte differentiation, SpheUSSC retained the ability for osteogenic differentiation. Under standard conditions for osteogenic differentiation, the first osteoblastic cells appeared at 70% confluency after 7 d and after 14 d all cells uniformly adopted an osteoblast-like cellular phenotype, which was verified by Alizarin Red S staining and detection of alkaline phosphatase mRNA, a marker of mature osteoblasts (Figure 3).

### 3.3. SpheUSSCs Contain a Uniformly Patterned, Partially Methylated OCT4 5′ Region and Expression of OCT4

**Figure 4:**
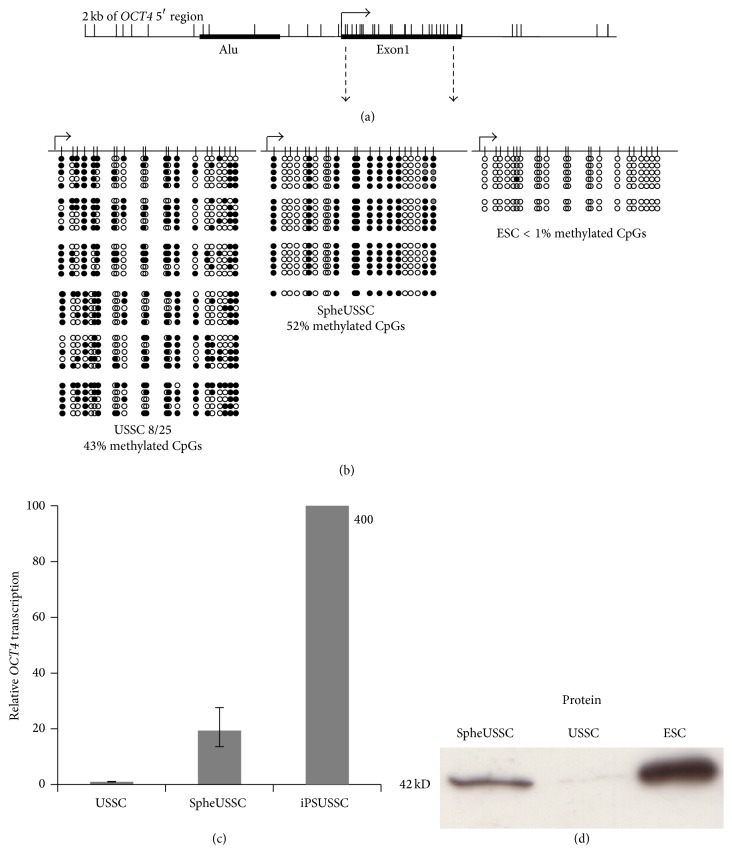
DNA methylation of* OCT4* 5′ region and OCT4 expression in USSCs 8/25 and SpheUSSCs. On top a schematic outline of the genomic organization of* OCT4* 5′ region is shown which includes the relative position of an Alu element, the transcription start site (solid arrow), first exon, and CpG dinucleotides (vertical bars) (a). Dashed arrows demarcate the 469 bp region analyzed by bisulfite genomic sequencing. DNA methylation patterns of USSCs 8/25, SpheUSSCs, and ESC are shown (b). Blank circles indicate unmethylated CpG dinucleotides and filled circles indicate methylated CpG dinucleotides. In addition transcription of* OCT4* as has been relatively quantified in USSCs, SpheUSSCs, and iPSUSSCs is shown in (c) and OCT4 protein as has been detected by western blotting in USSC, SpheUSSC, and in addition in ESC-H9 reference cells is depicted (d).

USSCs do not express the master pluripotency regulator gene OCT4 [2] and the OCT4 5′ region is partially methylated [13]. We therefore wondered whether methylation in this OCT4 5′ region is altered by the 5-Aza-CdR treatment and SpheUSSCs express OCT4 as a consequence. Bisulfite genomic sequencing revealed that all SpheUSSCs have acquired an altered, uniformly patterned [19], with unmethylated areas scattered, OCT4 5′ region (Figure 4(b)). Compared to the USSC source cells, OCT4 mRNA and protein expression was enhanced in SpheUSSCs as shown by real-time reverse transcription PCR (RT-PCR) and western blotting (Figures 4(c) and 4(d)). The used antibody was raised against amino acids 1–134 of OCT4 of human origin, has no cross-reactivity with OCT4 isoform B, and was kindly provided by Dr. Holm Zaehres, MPI Münster. Thus our epigenetic treatment led to the activation of the master pluripotency regulator OCT4 in SpheUSSCs.

### 3.4. SpheUSSCs Do Not Express hTERT but Have Acquired Prolonged Telomeres

**Figure 5:**
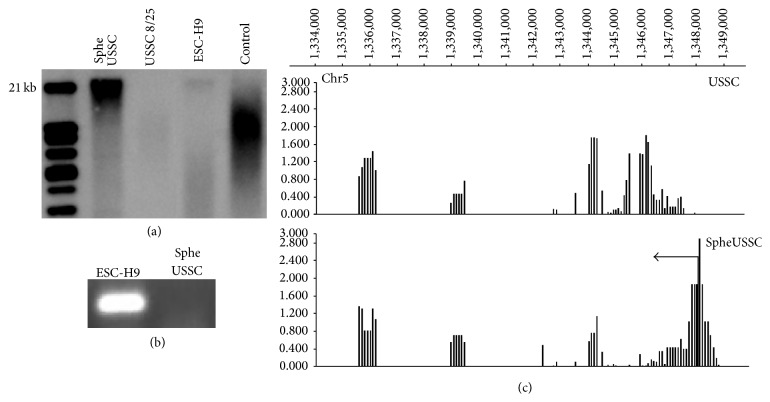
Telomere length and expression and DNA methylation of* hTERT* in SpheUSSCs. (a) Shows telomeres length of SpheUSSCs, USSC8/25, and ESC-H9, as detected by telomerase assay. (b)* hTERT* transcription analysed by conventional PCR in the ES cell line H9 and in SpheUSSCs is shown. DNA methylation patterns of the* hTERT* 5′ region of USSCs and SpheUSSCs as uncovered by NimbleGen analysis are shown in (c). The higher a bar is, the higher the DNA methylation is at the corresponding 75 bp long CpG rich DNA sequence.

As mentioned above, SpheUSSC did not show signs of proliferation decline or senescence over more than 150 passages, that is, far beyond the Hayflick limit [20]. We therefore wondered whether expression of the human telomerase gene hTERT or its DNA methylation status may have become affected and changed by our epigenetic treatment. A fundamental role of epigenetic regulation is well described for this gene [21]. Since the hTERT 5′ region has an unusual long CpG island, we first chose NimbleGen array technology to analyze the DNA methylation profile of 3.5 kb around the transcription start site of the hTERT gene. We found that the methylation pattern had been clearly changed in SpheUSSCs. In particular, a less methylated region encompassing the transcription start site in USSCs became clearly hypermethylated in SpheUSSCs (Figure 5(c)). For this region, a direct correlation between hypermethylation and gene expression has been demonstrated [21, 22]. Nevertheless, hTERT mRNA remained undetectable by RT-PCR in SpheUSSCs (Figure 5(b)). Surprisingly, the SpheUSSC telomeres were elongated up to 21 kB (Figure 5(a)). This finding that the telomeres of SpheUSSCs were significantly longer than those of the USSC 8/25 was verified by a second independent method [17] (supplementary data). Thus we found in SpheUSSC a situation where telomerase expression is absent and telomeres are elongated as it is known from early embryo cleavage stage, embryonic stem cells, induced pluripotent stem cells and cancer cells [23], despite the absence of telomerase. This is thought to be happening by telomerase independent, recombination-based mechanisms [24].

## 4. Discussion

Kögler et al. identified a rare, CD45 and HLA class II-negative stem cell candidate displaying robust in vitro proliferative capacity without spontaneous differentiation but with intrinsic and directable potential to develop into mesodermal, endodermal, and ectodermal cell fates. They named this primary population unrestricted somatic stem cell (USSC) [25]. Subsequently the same group classified CB-derived cells with high adipogenic differentiation potential as CB MSC. These cells were distinct from those expressing DLK-1 and never differentiated towards the adipogenic lineage. These cells were named USSCs [2].

In this study we present an elaborated epigenetic treatment protocol, applied on a well-characterized multipotent USSC line originated in human cord blood which led to the isolation of a new stable, stem cell type with altered multipotency, which we named SpheUSSC. This new cell type is characterized by immortalisation and a uniform phenotype with an extended differentiation spectrum in comparison to the cell type of origin. The uniform DNA methylation profile of the 5′ region of the OCT4 gene reflects on the molecular level the clonal character of the SpheUSSCs, in contrast to the source USSC population which has been shown to represent, epigenetically and phenotypically, a heterogeneous population [2, 13]. Since the used drugs cause DNA demethylation and histone acetylation, both well known to accompany dedifferentiation processes, we infer that the resulting cell type is dedifferentiated compared to the source cell. This notion is supported by the presence of the pluripotency marker OCT4, the lengthened telomeres, and the immortal character of the cells and finally by the ability of SpheUSSCs to uniformly form well differentiated adipocytes, in addition to retention of the ability to form osteoblasts at least as efficiently as the source USSCs which are unable to differentiate into adipocytes [2]. Thus in respect of this acquired ability the spectrum of multipotency has been affected. In addition, the stable and clonal character of the SpheUSSCs support the notion that the cells have been forced into a distinct dedifferentiation niche, an epigenetically canalized cellular phenotype, characterized by substantial cellular stability, instead of representing an intermediary differentiated stem cell type or a temporary artifactual state. Considering the way how we propagated the SpheUSSCs, that is, starting with single small cell colonies, together with the uniformly structured methylation pattern of the OCT4 5′ gene region, we propose the explanation: a few USSCs were affected by our epigenetic treatment to reprogram their epigenome to develop the observed stable SpheUSSC phenotype which has an acquired ability for stable high proliferation under these selecting cultivation conditions. However, the possibility that treatment with these epigenetic modifiers resulted in an enrichment of a very rare adipocyte progenitor population present within the parental cell population cannot be excluded.

It is intriguing that elongated telomeres were found in SpheUSSCs without hTERT expression. This phenomenon is known from early embryo cleavage stages, embryonic stem cells, induced pluripotent stem cells, and cancer cells [23] and is thought to be due to a telomerase independent, recombination-based mechanism termed alternative lengthening of telomeres (ALT) [24]. It likely involves homologous recombination [26] that is facilitated by a loss of CpG methylation in subtelomeric regions [27]. Therefore we presume that our demethylation treatment has induced telomere recombination events leading to telomere lengthening and immortalization.

We are aware that this new established, nontransgenic cell line, SpheUSSC, appears promising as a new multipotent stem cell model for research which possesses distinct robustness under cell cultivation conditions and as a stem cell source for cellular regeneration processes. We are currently working on detailed characterization of further differentiation properties of this new stem cell type. Furthermore, we speculate that further stem cells from umbilical cord blood or other stem cell sources could be forced or canalized, respectively, into similar changes of cellular heterogeneity and differentiation behavior by our present or an accordingly modified epigenetic protocol. These wide implications are the major driver for this paper. Next we plan to apply our protocol on all stem cell clones arising after prolonged incubation of whole cord bloods in cultivation flasks and to comparatively characterize in detail the full differentiation potential of their epigenetically reprogrammed descendants. Finally, we would like to mention the notion on our observations that it appears possible to induce cellular transition from one stable adult stem cell type to another, by application of sole epigenetic modifiers, abandoning transgenic material.

## 5. Conclusions

We present here a new stable and robust multipotent stem cell type, generated from human umbilical cord blood stem cells, solely by treatment with epigenetic modifiers. The new SpheUSSC line has promising properties as a stem cell model for research and for cell replacement therapies. We describe the epigenetic protocol which led to the development of the cell line, in order to enable investigations on whether this cellular transition of one stem cell type to another may also be applied to other stem cells. Presumably this may rather work on epigenetically related stem cell types. In terms of Waddington's famous metaphoric illustration of embryonic development as an epigenetic landscape one might depict our finding as a switch of a marble, switching its path to a neighboring groove by overcoming an epigenetic barrier.

## Supplementary Material

Supplementary data encompass:
a) Senescence-associated beta-galactosidase (SA-β-gal) assay on USSC SA 8/25 cells (P6) and SpheUSSC cells (P20).
b) Quantitative PCR measurement of absolute telomere length of USSC 8/25 and SpheeUSSC cells by O´Callaghan and Fenech method.
c) A table with all primers used in this study.

## Abbreviations

USSCs: Unrestricted somatic stem cells
ESC: Embryonic stem cells
iPSC: Induced pluripotent stem cells
5-Aza-CdR: 5-aza-2′-deoxycytidine
Aza: 5-azacytidine
TSA: Trichostatin A.
