# Supplementary material for: Effect on Multipotency and Phenotypic Transition of Unrestricted Somatic Stem Cells from Human Umbilical Cord Blood after Treatment with Epigenetic Agents
Source: Stem Cells Int. 2015 Dec 14;2016:7643218. doi: 10.1155/2016/7643218 (PMC4691642; doi:10.1155/2016/7643218)
Supplement: Supplementary file 1 — Supplementary data encompass: a) Senescence-associated beta-galactosidase (SA-β-gal) assay on USSC SA 8/25 cells (P6) and SpheUSSC cells (P20). b) Quantitative PCR measurement of absolute telomere length of USSC 8/25 and SpheeUSSC cells by O´Callaghan and Fenech method. c) A table with all primers used in this study. [file 7643218.f1.pdf]

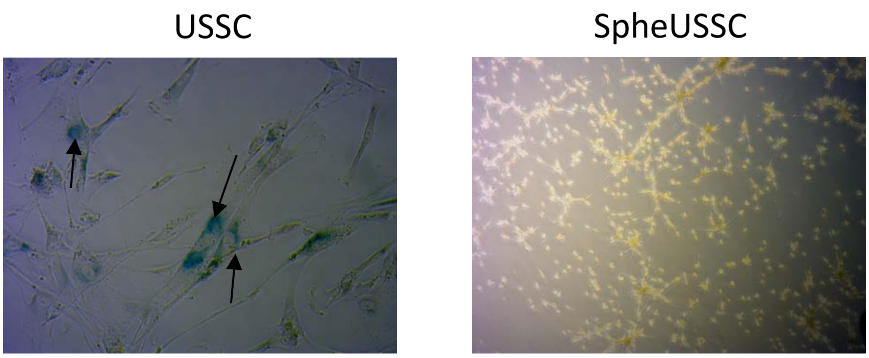

Fig. 1. Senescence-associated beta-galactosidase (SA-β-gal) assay on USSC SA 8/25 cells (P6) and SpheUSSC cells (P20). Blue-dyed precipitate characteristic of senescent cells is appearing only in USSC cells (arrows) but not in SpheUSSC cells.

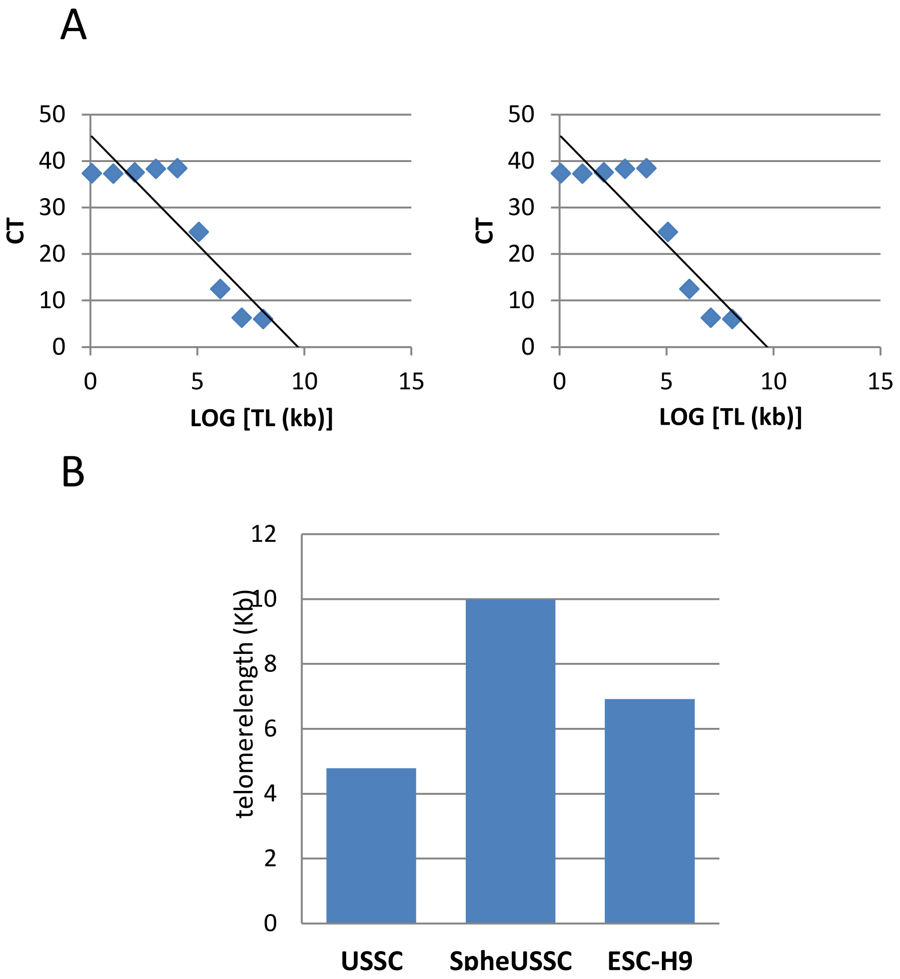

Fig. 2. Quantitative PCR measurement of absolute telomere length of USSC 8/25 and SpheeUSSC cells (see O´Callaghan and Fenech , 2011).

Standard curves used to calculate absolute telomere length. CT (cycle threshold) is the number of PCR cycles for which enough SYBR green fluorescence was detected above background. A) Graph shows standard curve for calculating length of telomere sequence per reaction tube. X-axis represents amount of telomere sequence in kb per reaction. The value generated from the experimental samples utilising this standard curve was equal to kb of telomere sequence per sample. B) Graph shows standard curve for calculating genome copies using 36B4 copy number. C) Diagram shows telomere lenght of USSC, SpheUSSC and ESC-H9 cells.

bisulfite genomic sequencing			
	sense primer	antisense primer	
OCT4konv	gagggagagaggggttgagtagtttt	actccaacttctccttctccaacttc	
relative transcription			
	sense primer	antisense primer	
OCT4	Gcccgaaagagaaagcgaaccag	tgacggagacagggggaaaggc	
SOX2	gcggcaatagcatggcgag	ccgaacccatggagccaag	
NANOG	cagcttccacgttgccttctgc	ccgaccacctcatctagccccc	
DNMT1	cttcttcagcacaaccgtcacc	ggtgcttgtccaggatgttg	
hTERT	agagtgtctggagcaagttgc	cgtagtccatgttcacaatcg	
DLK	gacccaccctgtgacccc	aggcagctcgtgcacccc	
LEPTIN	gccctatcttttctatgtcc	tctgtggagtagcctgaag	
ALPL	accaaagtctcgcgcccac	ccacggaggcttcagggc	
GAPDH	ttggaggcaaaaagctgactg	agagatatgccagtggcgttt	
telomere length			
	sense primer	antisense primer	
Telomerestandard	Ttagggttagggttagggttagggttagggttag gttagggttagggttagggttagggttagggttagggttagggttaggg		
36B4 standard	Cagcaagtgggaaggtgtaatccgtctccacagacaaggccaggactcgtttg acccgttgatgatagaatggg		
teloF	gtttgggtttgggtttgggtttggg tttgggtt		
teloR	ggcttgccttacccttacccttaccc ttacccttaccct		
36B4F	cagcaagtgggaaggtgtaatcc		
36B4R	cccattctatcatcaacgggtacaa		
b-globinF	gcttctgacacaactgtgttcactagc		
b-globinR	caccaacttcatccacgttcacc		
teloF	gtttgggtttgggtttgggtttggg tttgggtt		
teloR	ggcttgccttacccttacccttaccc ttacccttaccct		

Table. 1. All primers used in this study
